# The Role of Apelin in the Retina of Diabetic Rats

**DOI:** 10.1371/journal.pone.0069703

**Published:** 2013-07-16

**Authors:** Qiang Lu, Jing Feng, Yan-rong Jiang

**Affiliations:** 1 Department of Ophthalmology, People’s Hospital, Peking University, Beijing, China; 2 Key Laboratory of Vision Loss and Restoration, Ministry of Education, Beijing, China; NIAID, United States of America

## Abstract

**Purpose:**

Apelin is a novel adipocytokine participating in diabetes, but its role in diabetic retinopathy (DR) is unknown. Our study aimed to investigate the effect of apelin on the proliferative potential in DR along with its antagonist inhibitory effects.

**Principal Findings:**

Strong staining of apelin, co-localized with glial fibrillary acidic protein (GFAP) and vascular endothelial growth factor (VEGF) was observed in the retina of diabetic rats. Apelin, GFAP, and VEGF mRNA and protein levels were significantly increased in the sample’s retinas. Moreover, exogenous apelin promoted retinal Müller cell proliferation in vivo. Simultaneously, apelin induced GFAP and VEGF expression. F13A markedly reduced retinal gliosis caused by diabetes. Furthermore, F13A suppressed both GFAP and VEGF expression in vivo.

**Significance:**

Our results strongly suggest that apelin is associated with the development of DR and contributes to changes in the retinas of diabetic rats. Apelin induced promotion of cell proliferation lends support to the possibility that apelin may play a role in the progression of DR to a proliferative phase. This possible role deserves further investigation, which may offer new perspectives in the early prevention and treatment of DR.

## Introduction

Diabetic retinopathy (DR) is the leading cause of blindness in people of working age [1], [2]. It is caused by oxygen starvation in the retina, which induces glial cell damage and aberrant formation of blood vessels that destroy retinal architecture. Proliferative diabetic retinopathy (PDR), the final stage of DR, is characterized by abnormal fibrovascular proliferation and preretinal neovascularization induced by ocular ischemia with subsequent intravitreal hemorrhage and tractional retinal detachment [3]. VEGF has been considered to be the most important mediator of diabetic retinopathy [4]. Although inhibition of VEGF reduces retinal neovascularization, it does not completely inhibit ischemia-driven neovascularization and retinal cell proliferation. Thus, the involvement of other factors in this process seems likely.

Apelin is a peptide growth factor that binds the APJ receptor with high affinity [5]. Developmental studies have shown that apelin is highly expressed in many cells in the human body, including endothelium and vascular smooth muscle cells [6]–[8]. Moreover, some studies have shown that the apelin ligand is co-expressed with APJ in the developing blood vessels of perinatal mouse retina and glial cells, both in the brain and in the retina [9]–[12]. Recent studies have suggested that apelin contributes to the pathogenesis of diabetic retinopathy. Apelin was found to play an important role in obesity-related metabolic diseases [13]. Apelin inhibits insulin secretion in mice, which suggests a link between apelin and glucose homeostasis, and over-production of apelin in the obese is associated with obesity-related disorders such as type 2 diabetes [13]–[15]. The up-regulation of apelin in obesity can contribute to endocrine or metabolic dysfunctions, such as diabetic retinopathy [16]. In our previous study, we found that the concentration of apelin is higher in the vitreous of patients with PDR than in patients without diabetes. Apelin and APJ were positive expression in the fibrovascular membranes (FVMs) of patients with PDR [17], especially in glial cells and vascular endothelial cells; moreover, our research showed that anti-VEGF treatment cannot inhibit apelin expression and cannot completely suppress retinal neovascularization, which infers that apelin may play an important role in PDR and cannot be replaced by VEGF [18]. Apelin has various isoforms; the most widely studied are apelin-13 and apelin-36, with apelin-13 invariably exhibiting greater degrees of biological potency than apelin-36 [19]. All bioactivity is thought to reside in the terminal 13 aa fragment (apelin-13) [6]. The sequence of the 13 aa apelin peptide is 100% conserved between frogs and humans, suggesting that a critical function has been evolutionarily conserved [7].

Since apelin has been recognized as a factor contributing to diabetes and cell proliferation, and having not been investigated in DR, we propose a putative role for apelin in promoting proliferation in the retinas of those with DR. We conducted this study to investigate the effect of apelin and the treatment effect of its specific antagonist F13A on the retinas of diabetic rats in vivo.

## Materials and Methods

### Animals and Treatments

This study was approved by Peking University People’s Hospital Ethics Committee. Animals were purchased from the Laboratory Animal Center, Peking University People’s Hospital. Animal care and experiments were conducted under institutional guidelines and food and tap water were given ad libitum. Animals were sacrifice by cervical dislocation, in line with the euthanasia principle. Sixty overnight-fasted mature male Sprague-Dawley rats were given intraperitoneal injections of streptozotocin (STZ; 60 mg/kg body weight in 0.05 M sodium citrate buffer, pH 4.5; Sigma, USA) (Masuzawa et al., 2006). Blood glucose concentrations were measured from the tail vein using Medisafe mini GR-102 (Terumo, Tokyo, Japan). Diabetes was defined by blood glucose level of 300 mg/dl 48 h after the first injection of STZ. After 48 hours of successful modeling, which is giving the F13A intravitreal injection, blood glucose was measured within a month to maintain more than 300 mg/dl. The age-matched control group received the citrate buffer only. The rats were fed standard laboratory chow and allowed free access to water in an air-conditioned room with a 12 h light–dark cycle. After 12 weeks of treatment, rats were sacrificed under anesthesia and the retinas were harvested.

With the aid of a Hamilton microsyringe, apelin protein (10 ng/ml/eye; HD Bioscience, China) was carefully injected into left eyes of 30 normal rats. The injections were performed with the rats under light sodium pentobarbital anesthesia, with pupils dilated and using a dissecting microscope. The injected eyes were randomly assigned to the following post-injection times: 0, 3, 7, 14, and 30 days. Lastly, to evaluate the protective effect of F13A on the retinas of STZ-induced diabetic rats, F13A (20 ng/ml/eye; Phoenix Pharmaceuticals, Burlingame, CA) was carefully injected into right eyes of 20 diabetic rats. After injection, the eyes were inspected for hemorrhages, and if bleeding had occurred or if the lens capsule had been ruptured, that animal was not included in the study. Vehicle-treated animals received a single injection of phosphate-buffered saline.

### Immunofluorescence Staining

The eyes were enucleated and embedded in an optimum cutting temperature compound (Miles Laboratories, Naperville, IL), flash-frozen in liquid nitrogen, and then stored at −80°C. To detect apelin, immunohistochemistry frozen sections were cut by a cryostat (5 µm thick) and mounted on 3-aminopropyltriethoxysilane-coated glass slides. The tissue sections on the glass coverslips were fixed with 4% paraformaldehyde in phosphate-buffered saline (PBS; 20 min), washed (3×, PBS). Blocking was performed with 10% goat serum (1 h, 23°C). Primary antibodies were diluted into 10% goat serum/PBS and incubated overnight at 4°C. The following antibody was used: rabbit anti-apelin (1∶200, No. ab59469; Abcam, Cambridge, MA, USA). For double-labeling immunofluorescence study, they were then incubated with a monoclonal mouse anti-GFAP antibody (1∶250; Zhongshan Goldenbridge Biotechnology co.,Ltd, Beijing, China), and a monoclonal mouse anti-VEGF antibody (1∶200; No. sc-7269; Santa Cruz, USA). After blocking, the sections were washed (3×, PBS) and then incubated with secondary antibodies (1 h, 37°C). The secondary antibodies used were FITC-conjugated goat anti-mouse-tetramethyl rhodamine isothiocyanate (1∶200; No. ZF-0312; Zhongshan Golden bridge Biotech Co. Ltd, Beijing, China) and cyanogen Cy-3-conjugated goat anti-rabbit-fluorescein isothiocyanate (1∶200; No.BA1032, Sigma, USA). The samples were counterstained with DAPI (1∶1000; No.D9542, Sigma, USA) and covered by a non-fluorescent sealant; immunofluorescence was viewed using a fluorescence microscope (DS-Ri1-U2, Nikon, Japan) and images were acquired using a DS-U2u camera with NIS-Elements Imaging software. The expression of GFAP in each sample was quantified by densitometric scanning.

### Real-time Quantitative PCR

Total RNA was extracted from the retinas with an extraction reagent (TRIZOL®; Invitrogen), and 2 µg of retina RNA was converted into cDNA in a total reaction volume of 25 µl, containing 1 µg Oligo (dT), 5 µl M-MLV 5×buffer, 1.25 µl dNTP, 25 units Recombinant RNasin® Ribonuclease Inhibitor, and 200 units M-MLV reverse transcriptase. The mixture was incubated for 60 min at 42°C and terminated by heating at 95°C for 5 min. The target gene primers used for detection of apelin, GFAP, VEGF, and GAPDH are as follows, with the expected product size listed in base pairs (bp): rat apelin, sense,5′-GGCTAGAAGAAGGCAACATGC-3′ and anti-sense,5′- CCGCTGTCTGCGAAATTTC-3′, 101 bp; rat GFAP, sense, 5′-ACCATTCCGCGCCTCTCCCT-3′ and anti-sense, 5′-CTCAGCTGCCAGCGCCTTGT-3′, 204 bp; rat VEGF, sense,5′-AGAGCACCCTGCCCCTCTGG-3′ and anti-sense,5′-TGCCAGGACCCCTGTGGGAG-3′, 109 bp; and rat GAPDH, sense,5′-CCTGGAGAAACCTGCCAAGT-3′ and anti-sense,5′- TAGCCCAGGATGCCCTTTAG-3′, 83 bp. Real-time PCR assays were performed using IQ Supermix (Bio-Rad, Hercules, CA) with 20 µl reaction mixture containing 2 µl cDNA,7.2 µl sterilized water, 10 µl SYBR Green Real-time PCR Master Mix (2×), and 0.8 µl of each primer (10 µM). Amplification was carried out in 96-well plates on an iCycler iQ real-time detection system (Bio-Rad). Thermocycling conditions consisted of 3 min at 95°C for activation of the iTaq DNA polymerase and 35 cycles with a 95°C denaturation for 20 s, 63°C annealing for 15 s, and 72°C extension for 15 s. Each run included one synthetic template control and one no-template control for each target. Apelin, GFAP, and VEGF were normalized to GAPDH expression.

### Western Blot Analysis

Retinas were homogenized in 10 volumes of sample buffer (20 mM Tris-HCl, pH 7.4; 150 mM NaCl; 1 mM EDTA; 1% Triton X-100; 0.5% sodium deoxycholate; 0.1% SDS; 0.02% sodium azide; and 1 mM PMSF). The homogenates were incubated on ice for 30 min and were centrifuged at 12,000 rpm for 20 min at 4°C. Protein concentrations were determined on supernatants using the Bradford method protein microassay (Bio-Rad, Hercules, CA, USA). An equal amount of protein for each sample was heated to 100°C for 10 min with an equivalent volume of double strength sample buffer (containing 4% SDS and 10% mercaptoethanol) and loaded onto 15% polyacrylamide gels. The proteins were electrotransferred to a nitrocellulose membrane in Tris-glycine-methanol buffer and blocked for 1 h. The membrane was then incubated for 12 h at 4°C with a primary antibody against apelin, GFAP, VEGF, and β-actin (Sigma, USA). Then, after washing, the membrane was incubated with horseradish peroxidase-conjugated secondary antibody, rewashed, and processed for analysis, using an enhanced chemiluminescence (ECL) detection system (Amersham, Arlington Heights, IL, USA). Relative quantities were analyzed by densitometry using Bandscan 4.5 software.

### Statistical Analysis

Statistical analysis was performed using a commercially available statistical software package (SPSS for Windows, version 17.0, USA). The results were expressed as mean ± SD. Statistical differences were assessed using one-way analysis of variance (ANOVA); *p*<0.05 was considered statistically significant. Experiments were performed at least three times.

## Results

### Immunofluorescence Staining of Apelin, GFAP, and VEGF in the Rat Retinas

**Figure 1:**
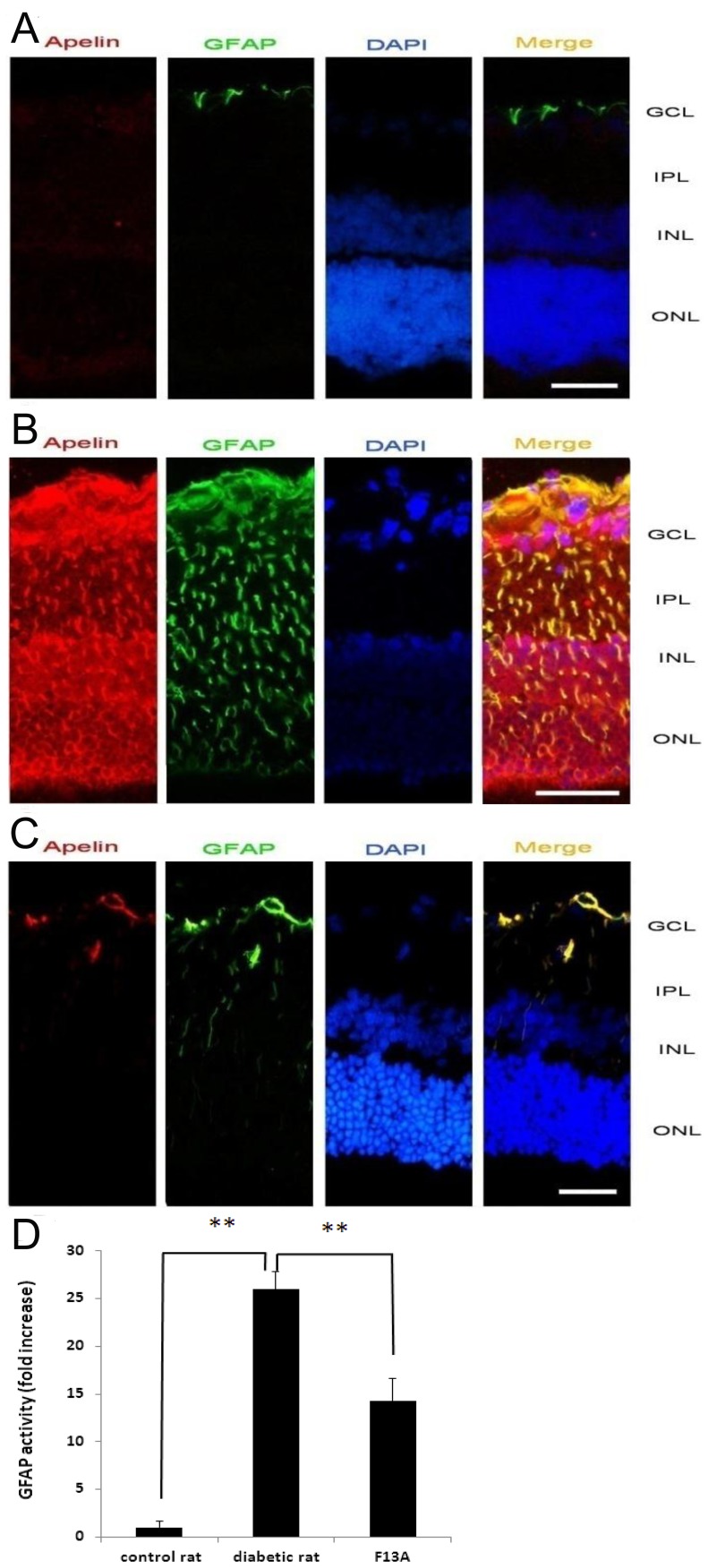
Indirect immunofluorescence evaluation of apelin and GFAP distribution in the retinas of normal rats (A), diabetic rats (B), and F13A-injected diabetic rats (C). Densitometric quantification of GFAP activity is shown (D). Cryosections adjacent to those presented in Figure 1 were probed with antibodies against apelin and GFAP and were detected using fluorochrome-conjugated secondary antibodies. Nuclei were detected using DAPI. Merged images contain three color channels representing apelin (red), GFAP (green), and DAPI (blue). GCL, ganglion cell layer; INL, inner nuclear layer; IPL, inner plexiform layer; ONL, outer nuclear layer; F13A, F13A-injected group. ** indicates *p*<0.01. Scale bars: 50 µm.

**Figure 2:**
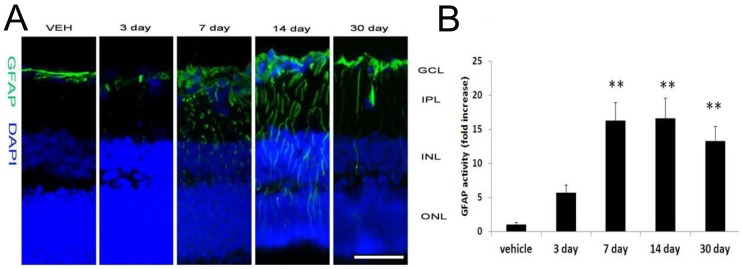
Effects of apelin protein on Müller cells in normal rats. Immunofluorescence evaluation of GFAP distribution in the retinas of rats after intravitreal injection of apelin is shown (A). Densitometric quantification of GFAP activity is shown (B). GFAP were detected using fluorochrome- conjugated secondary antibodies. Nuclei were detected using DAPI. Merged images contain two color channels representing GFAP (green) and DAPI (blue). GCL, ganglion cell layer; IPL, inner plexiform layer; INL, inner nuclear layer; ONL, outer nuclear layer; VEH, vehicle. ** indicates *p*<0.01 vs. vehicle. Scale bars: 50 µm.

In the retina of normal rats, staining of apelin was not found and GFAP was only expressed in the astrocytes of inner retinas (Fig. 1A), while there was significant enhancement of apelin and GFAP expression in the retinas of diabetic rats 12 weeks after STZ injection (Fig. 1B). Staining of apelin appeared over the whole vertical section of the retinas of diabetic rats, co-expression with GFAP, especially on Müller cells. Accompanied by marked gliosis, the number of Müller cells significantly increased with the duration of diabetes and Müller cell processes extending from the internal limiting membrane to the external membrane. Compared with diabetic rats, after injection of F13A, the expression of GFAP was decreased significantly (Fig. 1C). Densitometric semiquantification showed that the expression of GFAP was significantly decreased in the retinas of F13A-injected diabetic rats (*p*<0.01, Fig. 1D). To investigate whether apelin promotes cell proliferation, apelin was injected into the eyes of normal rats. Compared with the vehicle group, strong staining of GFAP was detected in glial cells, especially in Müller cells (Fig. 2A). Ganglion layer cells and Müller cells were either activated or proliferated 3 to 30 days after injection of apelin, and took 7 to 14 days to reach peak. The densitometric semiquantification showed that GFAP expression was significantly more increased than the vehicle group at 7, 14, and 30 days after apelin injection (*p*<0.01, Fig. 2B).

### mRNA and Protein Expression of Apelin, VEGF, and GFAP in vivo

**Figure 3:**
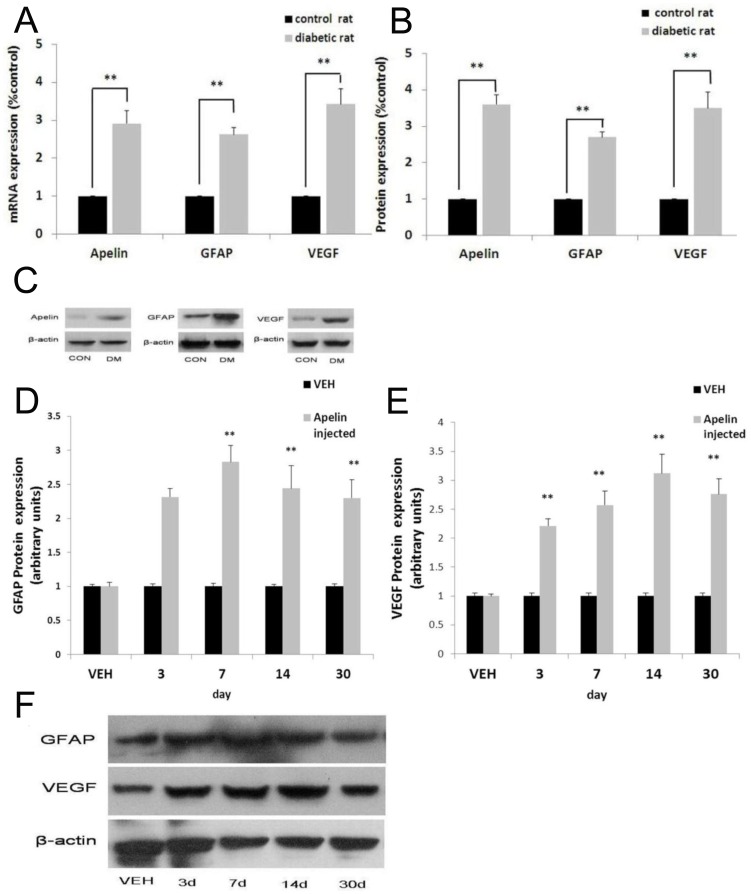
RT-PCR and Western blot analysis showed the mRNA (A) and protein (B) expression of apelin, GFAP, and VEGF in diabetic rats. Representative Western blots are shown (C). ** indicates *p*<0.01 vs. CON. The expression of GFAP (D) and VEGF (E) in the retinas of apelin injected rats, expressed as arbitrary units. Representative Western blots are shown (F). ** indicates *p*<0.01 vs. VEH.

RT-PCR showed that compared with controls (con), the expression of mRNA of apelinincreased 2.9-fold (*p*<0.01 vs. con, Fig. 3A), GFAP mRNA increased 2.6-fold (*p*<0.01, vs. con, Fig. 3A), and VEGF mRNA increased 3.4-fold (*p*<0.01, vs. con, Fig. 3A) in the retinas of diabetic rats 12 weeks after STZ-injection. Western blot analysis showed that the protein level of apelin, GFAP, and VEGF increased significantly compared with the control group. The expression of apelin protein (13 kDa) increased 3.6-fold (*p*<0.01, Fig. 3B), GFAP protein (56 kDa) increased 2.7-fold (*p*<0.01, Fig. 3B), and VEGF protein (21 kDa) increased 3.5-fold (*p*<0.01, Fig. 3B) in the retinas of diabetic rats compared with controls at 12 weeks. The molecular mass of β-actin (42 kDa) was used as a loading control. To investigate the effect of apelin protein on the expression of GFAP and VEGF in retinas, we injected apelin into eyes of normal rats. We found that the protein of GFAP (Fig. 3D) and VEGF (Fig. 3E) gradually increased, and GFAP expression peaked at 7 days and VEGF expression peaked at 14 days. Compared with the control group, both the expression of proteins and mRNA in the 7d ∼ 30d was significantly increased (*p*<0.01).

### Effect of Apelin Signaling Blockade on VEGF and GFAP Expression in Diabetic Rats

**Figure 4:**
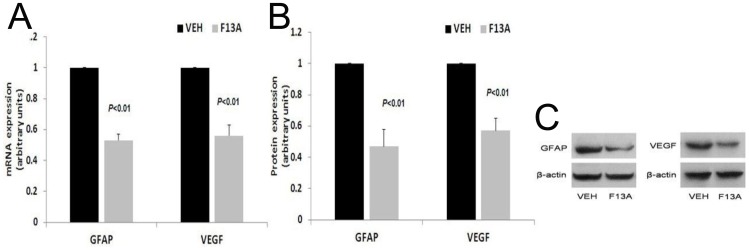
The effect of F13A on VEGF and GFAP mRNA (A) and protein (B) levels in the retinas of diabetic rats were analyzed using RT-PCR and Western blot in vehicle-treated diabetic rats (VEH) and F13A-treated diabetic rats (F13A). Representative Western blots are shown (C).

To evaluate the inhibitory effect of the apelin specific antagonist F13A on the retina of STZ-induced diabetic rats, we determined the expression of VEGF and GFAP proteins in the retinas from F13A-treated and vehicle-treated diabetic rats. Interestingly, treatment with F13A produced a marked reduction in the expression of VEGF mRNA (45% decrease; *p*<0.01) and GFAP mRNA (53% decrease; *p*<0.01), compared with the vehicle-treated diabetic rats (Fig. 4A). The expression of VEGF protein was decrease by 46% (*p*<0.01) and GFAP protein expression was decrease by 52% (*p*<0.01; Fig. 4B, C).

## Discussion

The results of this study show that strong staining of apelin, co-localized with GFAP and VEGF, was observed in the retina of diabetic rats. Apelin, GFAP, and VEGF mRNA and protein levels were significantly increased in the retinas of diabetic rats. Moreover, apelin activates signaling pathways in Müller cells and modulates Müller cell proliferation and GFAP and VEGF expression. Exogenous apelin promoted retinal Müller cell proliferation in vivo. Simultaneously, apelin induced GFAP and VEGF expression. F13A markedly reduced retinal gliosis caused by diabetes. Furthermore, F13A suppressed both GFAP and VEGF expression in vivo.

The present results were consistent with previous studies that suggested a relationship between apelin and cell proliferation [11], [12], [20], [21]. Some studies found that apelin is localized in glial cells [20]. Apelin mRNA was highly expressed in the nervous system, particularly in glial cells [11], [22], [23]. In addition, exogenous apelin could stimulate cell proliferation and inhibit apoptosis in glial and other cell lines [12], [24], [25]. One study found that with the increase of apelin concentration, the role of cell proliferation is enhanced. All these studies suggested that apelin has an irreplaceable role in cell proliferation and that the high expression of apelin under pathological conditions may be associated with the cell proliferation [26].

Furthermore, there were significantly higher mRNA and protein levels of GFAP and VEGF in the apelininduced group than in the non-apelininduced group, which supports the notion of the proliferative effect of apelin. To further ascertain whether the apelin signaling pathway modulates proliferation in DR, we performed inhibition experiments using a specific antagonist of apelin, known as F13A [27]. Notably, when blocking the effect of apelin with F13A, the expression of GFAP and VEGF is down regulated remarkably in diabetic rats. Moreover, the gliosis in the retinas of diabetic rats is suppressed. Such an expression pattern strongly argues in favor of the important role of apelin-mediated signaling in the modulation of cell proliferation throughout the progression of DR and suggests that apelin may associate with a progressive GFAP and VEGF overexpression. Our results are consistent with other studies showing that F13A led to a marked 52% decrease in splanchnic neovascularization and down-regulation of the expression of VEGF after induction of DR [28]. In addition, previous studies have reported that apelin and VEGF have positive synergistic effects, where increased expression of one can contribute to the expression of the other; i.e., VEGF and apelin show mutual promotion [29]. Some studies showed that knockout apelin or the APJ gene can inhibit the hypoxia-induced cell proliferation and this inhibition is not dependent on the VEGF signaling pathway [30]. Therefore, based on the present findings, it seems reasonable to assume that apelin may be involved in Müller cells proliferation during the development of DR.

Previous studies have suggested that Müller cells may be associated with retinal dysfunctions such as DR, proliferative vitreoretinopathy (PVR), and age-related macular degeneration (AMD) [31]–[33], and recent evidence indicated that DR also affects the Müller cells of the retina [34], [35]. Müller cells are an important constituent of the fibroproliferative scar tissue formed during PDR [36]. In agreement with previous reports, this study showed that the staining of GFAP (the marker of glial cells), co-localized with apelin, became positive expression in the retinas of diabetic rats and the elevation of apelin and GFAP in the retinas of diabetic rats [37]. This increase of apelin may due to the local production of apelin, presumably as an autocrine function of the retinal Müller cells. Therefore, the presence of apelin in Müller cells may suggest a putative role of this peptide on the proliferation states observed in the retinas of diabetic rats, and apelin production within the retina could easily exert effects locally, although it is yet unknown how, or even if, Müller cells release soluble apelin. Apelin is indeed a secreted protein that is expressed in cultured rodent hypothalamic, stomach, and breast cell lines [38]. The demonstration of the release of apelin would further support a role for apelin as an endogenous functional neuropeptide within the retina. Some studies have reported that Müller cells participate in the establishment of the blood–retinal barrier, which is constituted by the tight junctions between vascular endothelial cells [39]. A disturbance of this function is assumed to contribute to vessel leakage in DR. In vitro, Müller cells enhance the endothelial cell barrier function under normoxic conditions and impair the barrier function under hypoxic conditions [40]. In the present study, Müller cells were found to cause apelin to act on nearby vasculatures directly to respond to hypoxia of the inner layers, thereby inducing the formation of the superficial layer of vessels [41].

Finally, some studies have showed that apelin can be up-regulated by hypoxia, and the apelin signal pathway was shown to mediate hypoxia-induced cell proliferation [21], [30], [42]. HIF-1 could regulate and stimulate apelin gene expression [43], [44]. Some studies have indicated that apelin up-expression may protect nerve and endothelial cells from injury [12], [45]. Hypoxia is a strong stimulator of proliferation both during embryonic development and in pathological conditions such as malignant growth and DR by inducing the expression of several proliferative factors. Several reports have suggested that apelin is shown to be mitogenic and to possess chemotactic activity and antiapoptotic activity for cultured endothelial cells [26]. Furthermore, apelin induced by hypoxia was shown to have a proliferation activity for retinal endothelial and vascular smooth muscle cells, both in vitro and in vivo [16], [30]. Thus, hypoxia-induced apelin expression may provide a new mechanism involved in the adaptive physiological and pathophysiological response of Müller cells to low oxygen level, where hypoxic tissues drive the proliferation of glial cells in part by the secretion of apelin. One study proved that a positive feedback loop of regulation can exist where hypoxia induces apelin expression through HIF-1 and subsequently the mTOR pathway, which, in turn, activates HIF-1 and enhances hypoxia-induced apelin expression [30]. Taken together, these data suggest that apelin has the potential to modulate Müller cell proliferation in the mature organism and that hypoxic regulation of the apelin expression could play an essential role in DR.

Meanwhile, there are limitations to our study. Firstly, STZ-induced diabetic rats cannot correspond precisely to T2DM in human subjects, which is typically preceded by obesity [46]. Secondly, apelin is a novel adipokine participating in obesity-related metabolic diseases [13], [15]. Thus, it was studied by diet-induced obesity (DIO) models of type 2 diabetes, which may be better representative of the etiology of T2DM in humans. However, from our study, the high-level expression of apelin in vivo and the proliferative effect of apelin on Müller cells can be observed.

In conclusion, this study demonstrated that (i) the expression of apelin was increased in the retinas of diabetic rats in vivo; (ii) exogenous apelin could promote cell proliferation in vivo, up-regulate the retinal cell functions, and increase the expression of GFAP and VEGF; (iii) and apelin antagonist F13A protected Müller cells against hypoxia-mediated GFAP and VEGF expression and significantly down regulated functions of cell proliferation.

### Conclusion

Our results strongly suggest that apelin is associated with the development of DR and contributes to the changes of retinas in diabetic rats. Apelin induced promotion of cell proliferation lends support to the possibility that apelin may play a role in the progression of DR to a proliferative phase. This possible role deserves further investigation, which may offer new perspectives to the early prevention and treatment of DR [47]. It is likely that therapies that inhibit the pathway of apelin will form an important component of future therapy in patients with diabetes, acting in conjunction with conventional approaches to prevent DR.
